# Author Correction: Non-Abelian generalizations of the Hofstadter model: spin–orbit-coupled butterfly pairs

**DOI:** 10.1038/s41377-021-00519-4

**Published:** 2021-04-07

**Authors:** Yi Yang, Bo Zhen, John D. Joannopoulos, Marin Soljačić

**Affiliations:** 1 Department of Physics and Research Laboratory of Electronics, Massachusetts Institute of Technology, Cambridge, MA 02139 USA; 2 Department of Physics and Astronomy, University of Pennsylvania, Philadelphia, PA 19104 USA

**Keywords:** Physics, Optical physics

Correction to: *Light: Science & Applications*

10.1038/s41377-020-00384-7 published online 19 October 2020

Eq. (3) of the originally published paper:

“${\boldsymbol{A}} = \left( {0,m\theta \sigma _y + m\phi \sigma _z,0} \right)$” should be corrected to

“$${\boldsymbol{A}} = \left( {0,{\mathrm{log}}\left[ {\exp \left( {{\mathrm{i}}m\theta \sigma _y} \right)\exp \left( {{\mathrm{i}}m\phi \sigma _z} \right)} \right],0} \right)$$”

This has been corrected in both the PDF and the HTML versions of the paper.

