# Supplementary material for: Non-Abelian generalizations of the Hofstadter model: spin–orbit-coupled butterfly pairs
Source: Light Sci Appl. 2020 Oct 19;9:177. doi: 10.1038/s41377-020-00384-7 (PMC7572376; doi:10.1038/s41377-020-00384-7)
Supplement: Supplementary file 1 — Supplementary Information [file 41377_2020_384_MOESM1_ESM.pdf]

Supplementary Information

for

Non-Abelian Generalizations of the Hofstadter model: Spin-orbit-coupled Butterfly Pairs

Yi Yang[1],* Bo Zhen[2], John D. Joannopoulos[1], and Marin Soljačić[1]

[1] Department of Physics and Research Laboratory of Electronics, Massachusetts Institute of Technology, Cambridge, Massachusetts 02139, USA

[2] Department of Physics and Astronomy, University of Pennsylvania, Philadelphia, Pennsylvania 19104, USA

*Correspondence: Yi Yang (yiy@mit.edu)

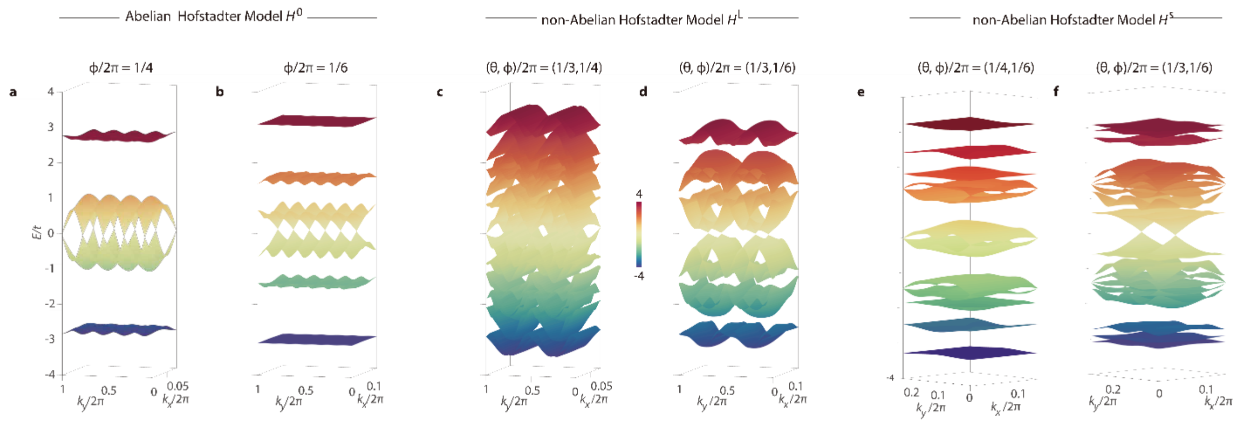

Figure S1. Full energy spectra for Fig. 3 in the main text, which focuses on the Weyl and Dirac points at zero energy.

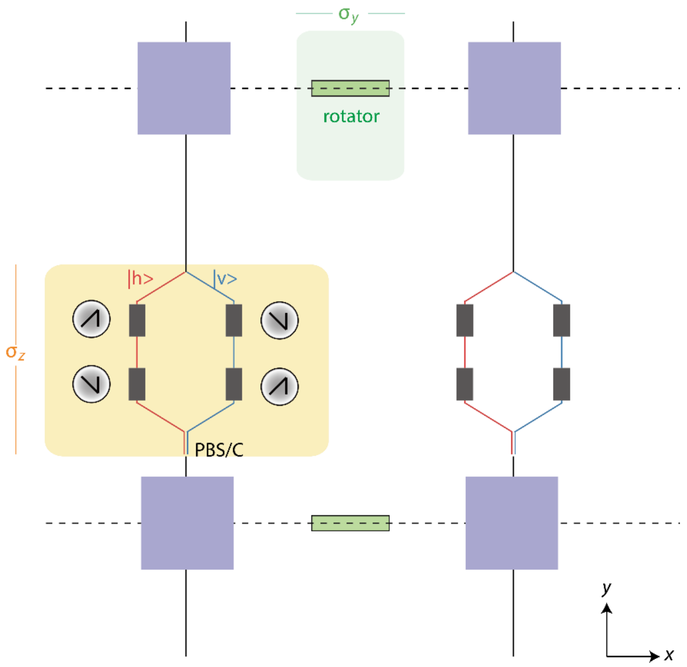

Figure S2. A possible coupled-resonator realization for the symmetric-gauge model H^S. Each resonator (purple), supporting two degenerate resonances of orthogonal polarizations, couples to adjacent resonators horizontally (dashed line) vertically (solid line) via the Faraday rotation and ($\theta\sigma_y$) dynamic modulation ($\phi\sigma_z$), respectively.With integrated photonics, the Faraday rotation could be realized with magneto-optical materials (green rectangle) and the dynamic modulation scheme could be realized with multiple lithium niobate phase modulators (grey rectangles). The detailed sawtooth modulation scheme follows that described in Ref. 46. PBS/C: polarization beam splitter and combiner.

Video 1 and 2: Bulk spectra animation for the Landau-gauge (video 1) and symmetric-gauge (video 2) models as a function of the amplitude of non-Abelian gauge fields. The Hofstadter butterfly re-emerges when the genuine non-Abelian condition is satisfied.
