# Supplementary figures and images for: Non-Abelian generalizations of the Hofstadter model: spin–orbit-coupled butterfly pairs
Source: Light Sci Appl. 2020 Oct 19;9:177. doi: 10.1038/s41377-020-00384-7 (PMC7572376; doi:10.1038/s41377-020-00384-7)

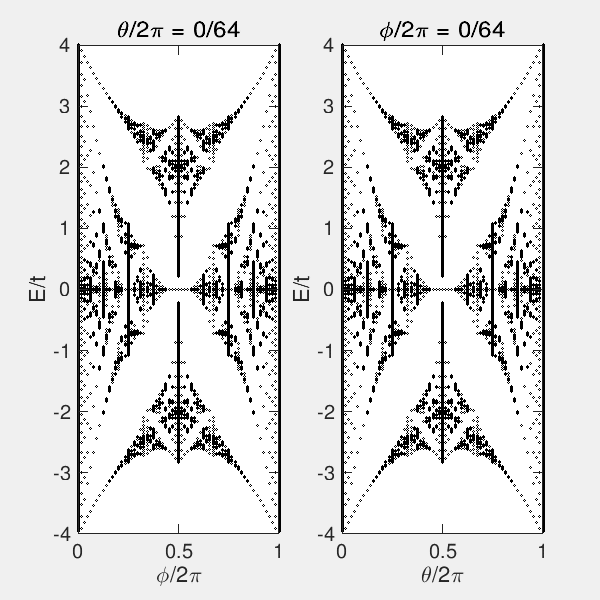

Supplement: Supplementary file 2 — Bulk spectra for the Landau-gauge model [file 41377_2020_384_MOESM2_ESM.gif]

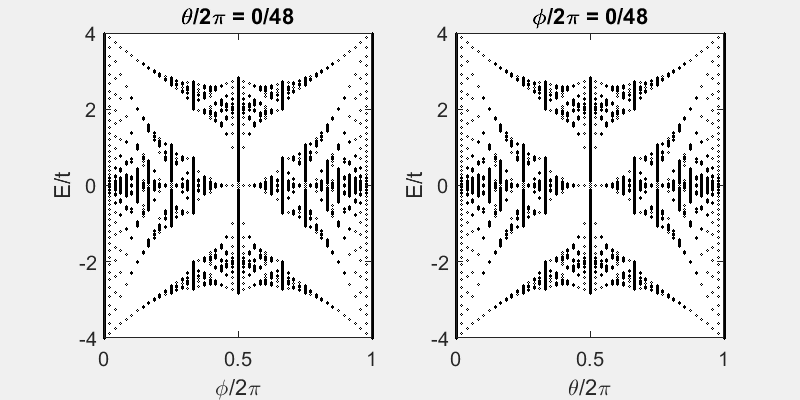

Supplement: Supplementary file 3 — Bulk spectra for the symmetric-gauge model [file 41377_2020_384_MOESM3_ESM.gif]
